# Neuroform Atlas stent-assisted coiling of wide-necked intracranial bifurcation aneurysms

**DOI:** 10.3389/fneur.2026.1737361

**Published:** 2026-02-11

**Authors:** Cheng Zhang, Yang Zhang, Chunwei Li, Shengli Shen, Hongzhou Duan

**Affiliations:** Department of Neurosurgery, Peking University First Hospital, Beijing, China

**Keywords:** endovascular treatment, intracranial bifurcation aneurysms, Neuroform Atlas stent, stent-assisted coiling, wide-necked

## Abstract

**Objective:**

To evaluate the safety and efficacy of Neuroform Atlas stent-assisted coiling in the endovascular treatment of wide-necked intracranial bifurcation aneurysms.

**Methods:**

We retrospectively reviewed 76 patients with wide-necked intracranial bifurcation aneurysms who underwent Neuroform Atlas stent-assisted coiling at our institution between August 2021 and December 2024. Patient demographics, aneurysm characteristics, procedural details, and procedure-related complications were collected and analyzed. Angiographic and clinical outcomes were assessed both postoperatively and during follow-up. The modified Rankin Scale (mRS) was used to evaluate clinical outcomes, and Raymond-Roy occlusion classification (RROC) grades were used to determine the degree of aneurysm occlusion.

**Results:**

A total of 76 patients harboring 81 wide-necked intracranial bifurcation aneurysms were included in the study. Among them, 17 patients (22.4%) presented with subarachnoid hemorrhage (SAH) at admission. A total of 87 Neuroform Atlas stents were successfully deployed in all patients. Immediate post-procedural angiography demonstrated complete occlusion in 69 aneurysms (85.2%, Raymond–Roy class I) and residual neck in 12 aneurysms (14.8%, Raymond–Roy class II). Procedure-related complications occurred in 4 patients (5.3%), including intraprocedural aneurysm rupture in 2 patients (2.6%), coil migration in 1 patient (1.3%), and parent artery hemorrhage in 1 patient (1.3%). Clinical follow-up was available for 72 patients (94.7%) at 6–18 months (mean, 12 months). At the last follow-up, 68 patients (94.4%) had an mRS score of 0, 3 patients (4.2%) had a score of 2, and 1 patient (1.4%) had a score of 3. A favorable clinical outcome (mRS ≤ 2) was achieved in 71 patients (98.6%), whereas 1 patient (1.4%) had a poor outcome (mRS>2). Angiographic follow-up was performed in 68 patients (89.5%) with 73 treated aneurysms (90.1%) at 6–18 months (mean, 12 months) after embolization. Complete occlusion (Raymond-Roy class I) was observed in 69 aneurysms (94.5%), and residual neck (Raymond-Roy class II) was found in 4 aneurysms (5.5%).

**Conclusion:**

Neuroform Atlas stent-assisted coiling is a feasible and effective endovascular treatment strategy for wide-necked intracranial bifurcation aneurysms. This technique is associated with high aneurysm occlusion rates, favorable clinical outcomes, and low procedure-related morbidity.

## 1 Introduction

Intracranial bifurcation aneurysms are located at arterial branch points exposed to high hemodynamic stress, most commonly at the middle cerebral artery bifurcation, basilar tip, anterior communicating artery, and internal carotid–posterior communicating artery junction (1). Most of these aneurysms have a wide neck (neck >4 mm or dome-to-neck ratio < 2), which makes endovascular treatment technically challenging (2). Coil embolization alone is often insufficient due to the risk of coil protrusion into the parent artery and inadequate aneurysm occlusion (3). Stent-assisted coiling provides mechanical support at the aneurysm neck, prevents coil herniation, and promotes progressive thrombosis by altering local hemodynamics (4–6).

The Neuroform Atlas stent is a low-profile, open-cell nitinol stent that can be delivered through a 0.0165-inch microcatheter, facilitating navigation in tortuous and distal vessels. It offers improved flexibility, conformability to the vessel wall, and scaffolding compared with previous stent designs. Although it has been increasingly used in clinical practice, evidence regarding its performance in wide-necked intracranial bifurcation aneurysms, particularly with long-term follow-up, remains limited. Therefore, this study aimed to evaluate the safety and efficacy of Neuroform Atlas stent-assisted coiling in the treatment of these complex aneurysms at a high-volume neurovascular center.

## 2 Materials and methods

### 2.1 Patient population

This retrospective study included patients with wide-necked intracranial bifurcation aneurysms who were treated in the Department of Neurosurgery, Peking University First Hospital, between August 2021 and December 2024. Inclusion criteria were as follows: (1) Age >18 years; (2) Diagnosis of wide-necked intracranial bifurcation aneurysm confirmed by digital subtraction angiography (DSA), computed tomography angiography (CTA), or magnetic resonance angiography (MRA), defined as neck diameter >4 mm or dome-to-neck ratio < 2; (3) Indication for aneurysm treatment, including ruptured aneurysm, symptomatic unruptured aneurysm, or aneurysms with large size or irregular morphology; (4) Informed consent for endovascular treatment obtained from the patient or their legal representative; (5) Treatment performed exclusively with the Neuroform Atlas stent; (6) Ability and willingness to undergo regular clinical and imaging follow-up. Exclusion criteria were as follows: (1) Preoperative Hunt–Hess grade V; (2) Severe cardiac or pulmonary dysfunction contraindicating anesthesia or intervention; (3) Allergy to contrast agents or metal materials; (4) Use of other stent types or intrasaccular devices (e.g., WEB); (5) Contraindications to antiplatelet therapy, such as gastrointestinal bleeding or hematologic disorders; (6) Contraindications to antiplatelet therapy, such as gastrointestinal bleeding or hematologic disorders.

### 2.2 Endovascular treatment procedure

All endovascular treatment procedures were performed under general anesthesia via femoral artery access. Following puncture of the right femoral artery, a 6F or 8F arterial sheath was introduced, and a guiding catheter was advanced into the internal carotid artery (ICA) or vertebral artery. Three-dimensional rotational angiography was performed to measure aneurysm diameter, neck width, parent artery diameter, and to determine the optimal working projection. An Echelon-10 microcatheter (Medtronic, Dublin, Ireland) was navigated into the aneurysm sac, while an Excelsior SL-10 or Echelon-10 microcatheter was positioned in the parent artery under microguidewire guidance. Stent-assisted coiling was primarily performed using the jailing technique. If coil herniation into the parent vessel was observed, a Neuroform Atlas stent was deployed across the aneurysm neck to secure coil placement. If, after deployment of the first stent, another branch vessel was considered at risk of occlusion during embolization, a second stent would be deployed using a Y-stenting technique. After deployment of the first stent, the initial microcatheter was often advanced along a shaped microguidewire, navigated through the struts of the first stent and positioned in the distal segment of the target branch. The second Neuroform Atlas stent was subsequently deployed in a Y-configuration. It should be pointed out that for lobulated aneurysms, embolization microcatheter can usually only be placed in one lobe, which will lead to the distribution of coils be often uneven. In this case, after releasing the Atlas stent, we usually shaped the microcatheter and placed it into another lobe of the aneurysm through the mesh of the Atlas stent. The aneurysm was finally densely occluded by zonal filling coils. Then the microcatheter was then withdrawn. Angiography via the guiding catheter was then performed to evaluate the grade of aneurysm occlusion and confirm the parent artery and its branches remain unaffected. A Dyna CT was performed to check that there was no new intracranial hemorrhage or ischemic stroke. Technical success was defined as the stent was deployed across the neck and the aneurysm was densely packed. The occlusion grade of intracranial aneurysm was evaluated based on DSA findings using the Raymond–Roy Occlusion Classification (RROC). Class I: complete obliteration; Class II: residual neck; Class IIIa: residual aneurysm with contrast within coil interstices; Class IIIb: residual aneurysm with contrast along aneurysm wall.

### 2.3 Antiplatelet regimen

For unruptured aneurysms, patients received dual antiplatelet therapy (DAPT) consisting of aspirin 100 mg/day and clopidogrel 75 mg/day for 5–7 days before endovascular treatment. All patients underwent testing of arachidonic acid (AA) inhibition rate and adenosine diphosphate (ADP) inhibition rate prior to endovascular treatment. An AA inhibition rate >50% and an ADP inhibition rate >30% were considered indicative of an adequate antiplatelet response. In patients with an inadequate response, clopidogrel was replaced with ticagrelor (90 mg twice a day). Postoperatively, DAPT was maintained for 3 months, followed by a single antiplatelet drug thereafter. For ruptured aneurysms, no preprocedural antiplatelet medication was administered. Instead, tirofiban was given intraprocedurally during stent deployment, with a bolus dose of 8.0 μg/kg infused intravenously over 3 min, followed by continuous infusion at 0.1 μg/kg/min for 24–48 h. A DAPT with bolus dosage (aspirin 300 mg and clopidogrel 300 mg bolus) was initiated on the first postoperative day, and then aspirin 100 mg/day and clopidogrel 75 mg/day continued for 3 months, followed by a single antiplatelet drug thereafter.

### 2.4 Postoperative evaluation and follow-up

Clinical follow-up was performed 3 months after discharge through telephone interviews or outpatient visits. The modified Rankin Scale (mRS) was used to evaluate clinical outcomes, with a good prognosis defined as mRS ≤ 2 and a poor prognosis as mRS >2. Digital subtraction angiography (DSA) was conducted approximately 12 months after the procedure to assess stent patency and aneurysm occlusion status (RROC classification).

### 2.5 Statistical analysis

Statistical analysis were performed using SPSS software (version 24.0; IBM Corp., Armonk, NY, USA). Continuous variables with a normal distribution were expressed as the mean ± standard deviation (SD), whereas those with a skewed distribution were expressed as the median with interquartile range (IQR). Categorical variables were presented as counts and percentages. Continuous variables were compared using the Student's *t*-test or Wilcoxon rank-sum test, as appropriate, and categorical variables were compared using the chi-square test or Fisher's exact test. A two-tailed *P* value < 0.05 was considered statistically significant.

## 3 Results

A total of 76 patients were included in the study, comprising 33 men and 43 women, with a mean age of 64.5 ± 10.5 years (range, 30–75 years; Table 1). Seventeen patients (22.4%) presented with subarachnoid hemorrhage (SAH), including five (6.6%) with Hunt–Hess grade I, ten (13.2%) with grade II, and two (2.6%) with grade III. The remaining patients had unruptured aneurysms. A total of 81 aneurysms were identified, with a mean maximum diameter of 6.0 ± 3.4 mm (range, 1.9–23.0 mm) and a mean neck width of 4.1 ± 1.5 mm (range, 1.8–11.0 mm). The aneurysms were located at the middle cerebral artery bifurcation in 42 (51.9%), basilar bifurcation in 10 (12.3%), anterior communicating artery in 16 (19.8%), and internal carotid artery bifurcation (posterior communicating artery) in 13 (16%).

**Table 1:** Baseline characteristics of patients and aneurysms.

**Variables**	**Data**
M/F	33/43
Age (y)	41–90 (64.5 ± 10.5)
No. of aneurysms (*n*)	81
Aneurysm size (mm)	1.9–23.0 (6.0 ± 3.4)
Aneurysm neck size (mm)	1.8–11.0 (4.1 ± 1.5)
MCA bifurcation (*n*, %)	42 (51.9%)
Basilar tip (*n*, %)	10 (12.3%)
Acom (*n*, %)	16 (19.8%)
ICA Pcom (*n*, %)	13 (16.0%)
SAH (*n*, %)	17 (22.4%)
Hunt–Hess I (*n*, %)	5 (29.4%)
Hunt–Hess II (*n*, %)	10 (58.8%)
Hunt–Hess III (*n*, %)	2 (11.8%)

*MCA*, middle cerebral artery; *Acom*, anterior communicating artery; *ICA*, internal carotid artery; *Pcom*, posterior communicating artery; *SAH*, subarachnoid hemorrhage.

**Figure 1:**
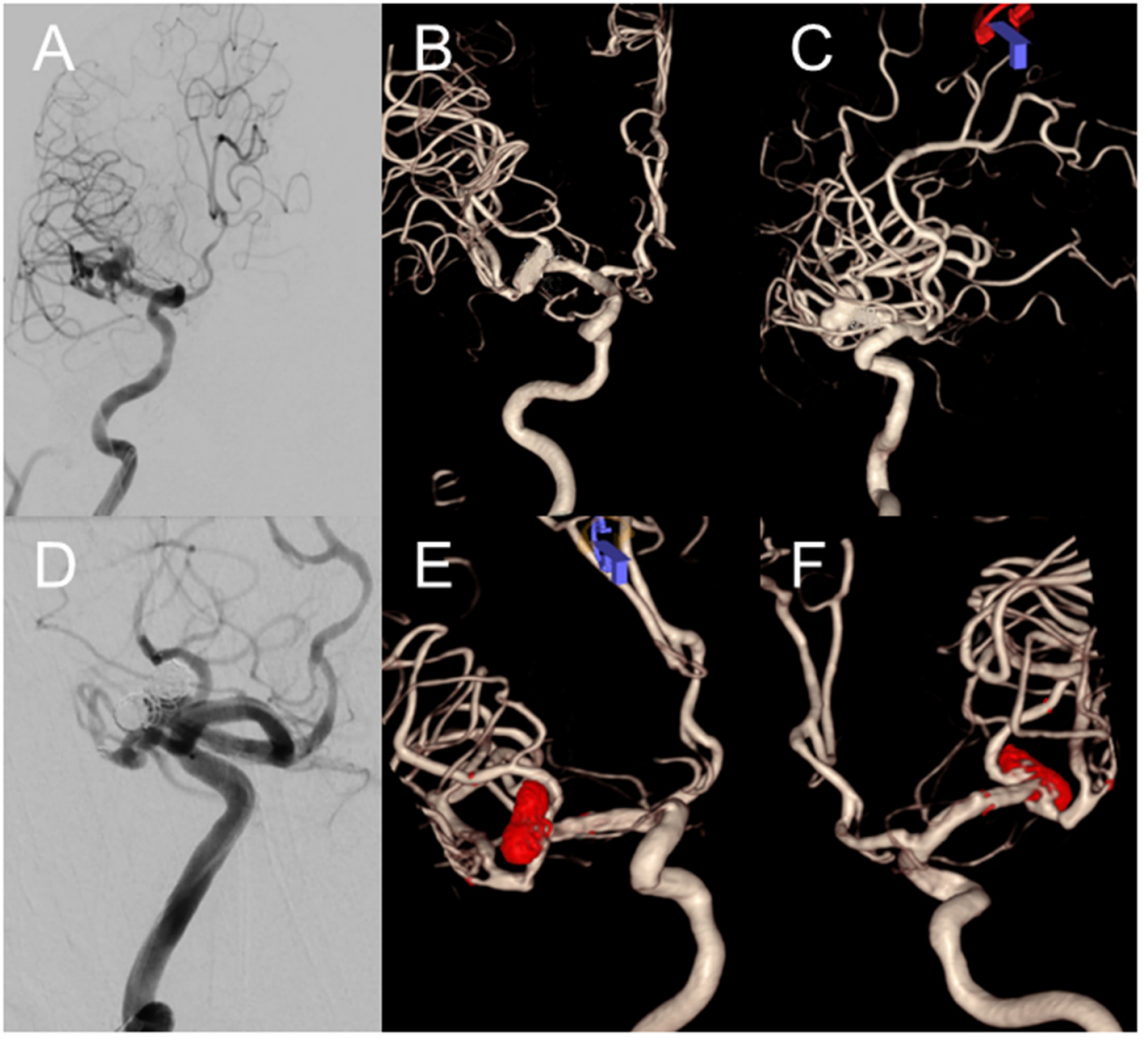
Images from a 73-year-old woman with a right middle cerebral artery (MCA) bifurcation aneurysm. Digital subtraction angiography with three-dimensional reconstruction demonstrates the right MCA bifurcation aneurysm **(A–C)**. Procedural angiography shows deployment of two Atlas stents into the superior and inferior divisions of the right M2 segment using a Y-stenting technique. The immediate post-procedural angiography reveals near-complete occlusion of the aneurysm with a minimal neck remnant **(D)**. Three-dimensional reconstruction images illustrate the radiopaque markers (red dots) at the proximal and distal ends of the Atlas stents, along with the coils within the aneurysm sac **(E, F)**.

The Neuroform Atlas stent was successfully deployed in all patients for the treatment of 81 aneurysms, with a total of 87 stents implanted (Table 2). Immediately after endovascular embolization, complete occlusion (Raymond–Roy grade I) was achieved in 69 aneurysms (85.2%), and residual neck (grade II) in 12 aneurysms (14.8%). There was no significant difference in the immediate occlusion rate between ruptured and unruptured aneurysms (*P* = 0.53; Table 3). A representative case is shown in Figure 1.

**Table 2:** Postprocedural and follow-up angiographic and clinical outcomes.

**Variables**	**Data**
No. of stents deployed (*n*)	87
**Raymond occlusion grades (** ***n*** **, %)**	
Grade I	69 (85.2%)
Grade II	12 (14.8%)
**Complications (** ***n*** **, %)**	
Aneurysm hemorrhage	2 (2.6%)
Coils displacement	1 (1.3%)
Parent artery hemorrhage	1 (1.3%)
**Clinical follow-up (** ***n*** **, %)**	
No. of patients	72 (94.7%)
mRS grade 0	68 (94.4%)
mRS grade 2	3 (4.2%)
mRS grade 3	1 (1.4%)
**Angiographic follow-up (** ***n*** **, %)**	
No. of patients	68 (89.5%)
Raymond grade I	69 (94.5%)
Raymond grade II	4 (5.5%)

mRS, modified Rankin Scale.

**Table 3:** Occlusion degrees of ruptured and unruptured aneurysms immediately after embolization.

**Variables**	**Total aneurysms (*n* = 81)**	**Unruptured (*n* = 64)**	**Ruptured (*n* = 17)**	***P***
No. of stents deployed (*n*)	87	67	20	NA
**Raymond occlusion grades (** ***n*** **, %)**				
Grade I	69 (85.2%)	59 (92.2%)	10 (58.8%)	0.53
Grade II	12 (14.8%)	5 (7.8%)	7 (41.2%)	

Periprocedural complications occurred in 4 patients (5.3%), including aneurysm rupture at the middle cerebral artery bifurcation in 2 patients (2.6%), coil displacement in 1 patient (1.3%), and hemorrhage from the parent artery of the anterior cerebral artery in 1 patient (1.3%). There was no significant difference in the incidence of periprocedural complications between ruptured and unruptured aneurysms (*P* = 0.45; Table 4). Clinical follow-up was available for 72 patients (94.7%) at 6–18 months (mean, 12 months) after embolization. At the last follow-up, 68 patients (94.4%) had an mRS score of 0, 3 patients (4.2%) had mRS 2, and 1 patient (1.4%) had mRS 3. No cases of delayed hemorrhage or ischemic events were observed. There was no significant difference in clinical outcomes based on mRS scores between ruptured and unruptured aneurysms (*P* = 0.45; Table 4).

**Table 4:** Complications and clinical prognosis in patients with ruptured and unruptured aneurysms.

**Variables**	**Total (*n* = 76)**	**Patients with unruptured aneurysms (*n* = 59)**	**Patients with ruptured aneurysms (*n* = 17)**	***P***
**Complications**				
Aneurysm hemorrhage	2 (2.6%)	2 (3.4%)	0 (0%)	0.53
Coils displacement	1 (1.3%)	1 (1.7%)	0 (0%)	
Parent artery hemorrhage	1 (1.3%)	0 (0%)	1 (5.9%)	
Clinical prognosis at follow-up	*n* = 72	*n* = 55	*n* = 17	
mRS grade 0	68 (94.4%)	54 (98.2%)	14 (82.3%)	0.45
mRS grade 2	3 (4.2%)	1 (1.8%)	2 (11.8%)	
mRS grade 3	1 (1.4%)	0 (0%)	1 (5.9%)	

Digital subtraction angiography (DSA) was performed in 68 patients (89.5%), covering 73 aneurysms (80.6%), at 6–18 months after embolization (mean, 12 months). At follow-up, complete occlusion (Raymond-Roy grade I) was observed in 69 aneurysms (94.5%), and residual neck (grade II) in 4 aneurysms (5.5%). There was no significant difference in the Raymond-Roy occlusion grades between immediate post-procedural and follow-up angiographic results (*P* = 0.50). No cases of in-stent stenosis or occlusion were detected during follow-up.

## 4 Discussion

The management of wide-necked intracranial bifurcation aneurysms remains challenging. The commonly treatment strategies include microsurgical clipping and endovascular embolization. Although clipping provides durable aneurysm occlusion, it carries substantial surgical risk, particularly in elderly patients or those with poor general health who may not tolerate general anesthesia (7). Endovascular embolization has become an important alternative; however, the complex vascular anatomy of bifurcation aneurysms often increases the risk of parent artery stenosis and procedural complications, achieving a lower complete occlusion rate compared with sidewall aneurysms (8). As each branch vessel at the bifurcation can't be sacrificed and most of these aneurysms are wide-necked, stent-assisted coiling is frequently required to achieve stable and safe aneurysm occlusion (9).

Several kinds of stents are available for stent-assisted embolization of bifurcation aneurysms, each with specific strengths and limitations. The LVIS stent is a braided, closed-cell device with higher metal coverage, offering enhanced neck scaffolding and flow-diverting effects. However, its denser mesh may limit microcatheter re-crossing after deployment and increase technical difficulty in bifurcation settings, especially when Y-stenting is required. The Enterprise stent is a closed-cell, retrievable device that provides good radial support but requires a larger 0.021-inch microcatheter, which may hinder navigation into sharply angled branch vessels and restrict its use in small or distal bifurcations. Solitaire AB, originally designed as a thrombectomy device, can be deployed in bifurcation aneurysms and offers retrievability; however, its relatively low metal coverage may reduce long-term aneurysm occlusion durability. A study by Lv et al. (10). included 20 patients with wide-necked aneurysms treated with Solitaire AB stent-assisted coiling, all of whom achieved adequate occlusion of aneurysms without ischemic or hemorrhagic complications. Follow-up angiography at 6 months showed 16 (80%) aneurysms were absolutely occluded.

There are several technical advantages of Atlas stent in the treatment of wide-necked bifurcation aneurysms. Its low-profile design, high trackability, and strong conformability to the vessel wall allow for reliable navigation through tortuous intracranial vasculature (11). The hybrid-cell configuration, featuring an open-cell design distally and a closed-cell design proximally, improves microcatheter stability during trans-cell access and enhances stent apposition. With a relatively low metal surface coverage of 6–12%, the Atlas stent demonstrates accurate positioning and minimal foreshortening after deployment. Compatibility with a 0.0165-inch microcatheter further expands its applicability in complex anatomy (12). The stent is suitable for parent vessels ranging from 2.0 to 4.5 mm in diameter (13, 14). Recent meta-analyses have supported the safety and effectiveness of the Atlas stent in both ruptured and unruptured aneurysms. Lynch et al. (15). Reported an adequate occlusion rate of 94.8% among 593 treated aneurysms, while Akram et al. (16) demonstrated a 95% adequate occlusion rate at follow-up in a cohort of 2,434 aneurysms. These findings suggest that the Atlas stent may provide favorable angiographic and clinical outcomes in complex bifurcation aneurysm treatment.

Various endovascular techniques have been introduced for wide-necked bifurcation aneurysms, including horizontal stenting and X- or Y-stenting configurations. Among these, Y-stenting is considered a viable option for managing complex bifurcation anatomy (17). One of the primary challenges of Y-stent coiling is microcatheter navigation into sharply angulated side branches (18). Early-generation Y-stents required delivery through 0.027- or 0.021-inch microcatheters, which posed technical difficulties due to their relatively large profiles and limited trackability, resulting in lower procedural success rates. The Neuroform Atlas stent, a successor to the original Neuroform design, is compatible with a 0.0165-inch microcatheter, facilitating safer and more controlled navigation within small or tortuous vessels. A second technical challenge in Y-stent coiling involves catheterizing the contralateral branch through the stent struts, which may lead to displacement of the first stent. The Atlas stent features a laser-cut hybrid-cell architecture, consisting of an open-cell mid-distal portion and a closed-cell proximal end. This configuration improves microcatheter support and allows smoother trans-cell access while reducing the risk of stent deformation or dislocation. Following Y-stent deployment, re-accessing the aneurysm sac can also be difficult. To mitigate this, the jailing technique—placing the microcatheter into the aneurysm before deploying the first stent—is often utilized to maintain stable access for coil delivery.

In general, the immediate occlusion outcomes following Y-stent–assisted coiling are favorable. Ciccio et al. (4). reported an adequate occlusion rate of 95% at follow-up in a series of 52 aneurysms treated with Y-configuration Atlas stents. Similarly, Aydin et al. (19). described 30 aneurysms treated with Y-stenting technique, noting an occlusion rate of 83.3% immediately following coil embolization and 93.3% at follow-up. Kim et al. (20). successfully treated 15 intracranial bifurcation aneurysms using Y-stenting with the Atlas platform, achieving immediate adequate occlusion in 46.7% of cases, neck remnant in 13.3%, and incomplete occlusion in 40%; at follow-up, occlusion rate improved to 73.3%. Furthermore, a meta-analysis by Cagnazzo et al. (21), involving 750 aneurysms treated with Y-stenting, demonstrated a long-term adequate or near-adequate occlusion rate of 95.4%, reinforcing the durability and effectiveness of Y-stent constructs for complex bifurcation aneurysms.

The vault technique is another commonly utilized strategy during Atlas stent deployment, particularly in selected wide-necked bifurcation lesions. By intentionally allowing controlled protrusion of stent struts toward the aneurysm neck during deployment, this technique enhances neck coverage while preserving branch vessels originating near the aneurysm orifice. Clinically, the vault technique may reduce the need for complex multi-stent configurations, thereby simplifying the procedure and potentially lowering procedural risks.

The vault technique is best suited for aneurysms located along the greater curvature of the parent artery, where stent struts can conform to the vessel wall and provide stable scaffolding against coil prolapse. It is especially useful for small or medium-sized wide-necked aneurysms in which preservation of branch vessels is critical. From a technical perspective, appropriate stent sizing is essential; selecting a larger-diameter stent, such as a 4.5-mm Neuroform Atlas stent, facilitates adequate strut expansion and vault formation. Controlled forward tension during stent deployment allows optimal cell opening without compromising stent stability. Careful angiographic assessment during deployment is required to avoid excessive protrusion or malapposition (22). Braided stents, such as the low-profile visualized intraluminal support (LVIS) device (MicroVention, Tustin, CA, USA), may achieve similar effects using the barrel or bulging technique (23, 24). Importantly, these techniques are most effective along the greater curvature of the parent artery, where stent struts conform well to the vessel wall and provide protection against coil protrusion. In the present study, the vault technique was applied in selected wide-necked bifurcation aneurysms, allowing single-stent–assisted coiling and reducing the need for more complex multi-stent configurations.

Complications associated with endovascular embolization of intracranial aneurysms primarily include intraprocedural aneurysm rupture, thromboembolic events, coil prolapse, and stent displacement. In a prospective, multicenter, single-arm study of 182 patients with wide-necked anterior circulation aneurysms, Zaidat et al. (25) reported an ipsilateral stroke rate of 4.4% following Atlas stent deployment. A subsequent European prospective multicenter trial involving 106 patients treated with at least one Atlas stent demonstrated an overall complication rate of 5.7% (26). Additionally, Jankowitz et al. (27). reported outcomes from 116 patients with unruptured, wide-necked posterior circulation aneurysms treated with Atlas assistance, achieving complete occlusion in 85.3% of cases, with ipsilateral stroke occurring in 4.3%. In comparison, the complication rate in our cohort was relatively low at 5.3%, including two cases of intraoperative aneurysm rupture (2.6%), one case of coil migration (1.3%), and one case with parent artery injury (1.3%).

In our study, dual antiplatelet therapy was administered to patients with unruptured aneurysms for at least 5 days before stent-assisted coiling and continued for 3 months postoperatively, after which a single antiplatelet drug was maintained. This regimen is widely accepted as the standard protocol to prevent thromboembolic events in stent-assisted coiling (28–30). Owing to the low metal surface coverage and excellent wall apposition of the Atlas stent, a shorter duration or lower intensity of antiplatelet therapy may be sufficient compared with other stents. For patients with ruptured aneurysms, preoperative antiplatelet therapy was not indicated. Instead, tirofiban was used intraoperatively immediately after stent deployment, followed by a loading dose of dual antiplatelet therapy with aspirin and clopidogrel in the next 24h. Tirofiban provides rapid platelet inhibition and effectively reduces thromboembolic risk in the acute setting (31, 32). Hou et al. (33). suggested that for ruptured aneurysms, a loading dose of aspirin (300 mg) and clopidogrel (300 mg) at least 3 h before the procedure may also be effective. Dual antiplatelet therapy should be maintained for at least 3 months after the procedure, followed by a single antiplatelet therapy thereafter. When two Atlas stents are deployed, extending the time of dual antiplatelet therapy to 6 months is recommended before transitioning to a single antiplatelet therapy.

### 4.1 Limitations

There are also some limitations in our study. Firstly, its retrospective, single-center design without randomization or a control group may introduce selection bias. Secondly, the relatively small sample size and limited follow-up duration restrict the generalizability of the findings and may underestimate delayed complications or recurrences. Thirdly, the relative small sample size (76 patients and 81 aneurysms) may underpower the study to perform meaningful subgroup analyses. Finally, the mean follow-up of 12 months is relatively short for evaluating long-term durability, stent-related stenosis, or delayed recurrences. Therefore, a prospective, randomized, multicenter trial with larger cohorts and longer follow-up should be warranted to validate our findings in future.

## 5 Conclusion

In conclusion, Atlas stent-assisted coiling of wide-necked intracranial bifurcation aneurysms is safe and effective. Outcomes and occlusion rates are favorable and morbidity is acceptable. Prospective multicenter studies with long-term follow-up are warranted to further validate the durability of aneurysm occlusion and long-term clinical outcomes.

## Data Availability

The raw data supporting the conclusions of this article will be made available by the authors, without undue reservation.
